# Supplementary material for: Macrophage spatial heterogeneity in gastric cancer defined by multiplex immunohistochemistry
Source: Nat Commun. 2019 Sep 2;10:3928. doi: 10.1038/s41467-019-11788-4 (PMC6718690; doi:10.1038/s41467-019-11788-4)
Supplement: Supplementary file 1 — Supplementary Information (Table and Figures) [file 41467_2019_11788_MOESM1_ESM.pdf]

Supplementary Information

Macrophage Spatial Heterogeneity in Gastric Cancer Defined by Multiplex Immunohistochemistry

Huang et al.

Supplementary Table 1. Clinical and pathological characteristics of the MAUGIC study cohort

Groups		Patient number	Percentage in cohort (%)
Age	90-99	1	1.79
	80-89	6	10.71
	70-79	16	28.57
	60-69	12	21.43
	50-59	16	28.57
	40-49	5	8.93
Gender	Female	20	35.71
	Male	36	64.29
Histological classification (Lauren)	Intestinal	37	66.07
	Diffuse	18	32.14
	Mixed	1	1.79
Molecular subtype (TCGA)	EBV	4	7.14
	MSI	9	16.07
	GS	17	30.36
	Others	26	46.43
T stage	T4	1	1.79
	T3	39	69.64
	T2	13	23.21
	T1	3	5.36
N stage	N3	5	8.93
	N2	10	17.86
	N1	18	32.14
	N0	23	41.07
M stage	M1	3	5.36
	M0	53	94.64
AJCC (6th)	4+3	27	48.21
	2+1	29	51.79
Recurrence	Yes	30	53.57
	No	24	42.86
	ND	2	3.57

ND: not documented.

Supplementary Figure 1

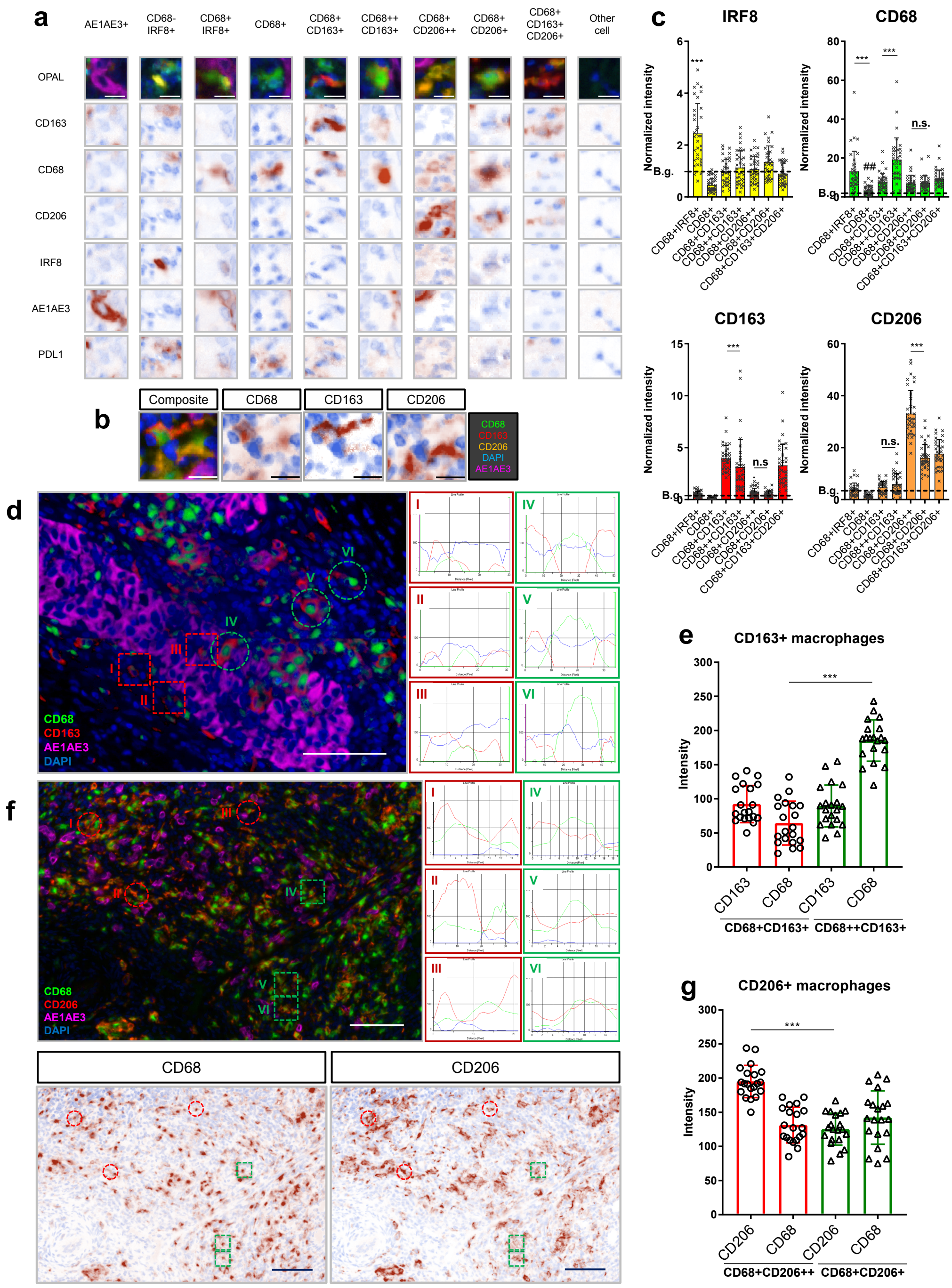

Supplementary Figure 1: Characterization of macrophage populations
a Representative images of the seven major TAM populations. Key: Yellow: IRF8, Green: CD68, Red: CD163, Orange: CD206, Magenta: AE1AE3, Cyan: PDL1, Blue: DAPI. Single-stained IHC color: Blue: DAPI, Brown: Positive staining of markers as indicated (+). Relative intensity between cell types is represented (++). Scale bar: 10 μm. **b** Representative images of adjacent CD163+ (CD206-) and CD206+ (CD163-) macrophages. Scale bar: 10 μm. **c** Marker signatures used for TAM population characterization in patient samples (n=35). Normalized intensity: original intensity of the marker / exposure time. B.g (dash line): background level of staining determined by the mean intensity of tumor (AE1AE3+) and non-macrophage (Other) cells. Error bars represent mean ± SD, ***p<0.001, n.s. not significant. Mann–Whitney's U test. ##p<0.01 comparing to tumor and non-macrophage cells. **d** Intensity of CD68 and CD163 on TAMs analyzed with the ImagePro software. Intensity of markers on each numbered cells were plotted in the matched numbered plots. Red square: CD68+CD163+ TAMs, Green circle: CD68++CD163+ TAMs. X axis: Distance in pixels, Y axis: Intensity of marker. Colors in the plots: CD163 (red), CD68 (green) and DAPI (blue). Scale bar: 100 μm. **e** Quantification of the maximum intensity of CD68 and CD163 expressed on the TAMs (n=20 for each population) in Supplementary Fig. 1d. Error bars represent mean ± SD, ***p<0.001. Mann–Whitney's U test. Each point represents one patient. **f** Intensity of CD68 and CD206 on TAMs analyzed with the ImagePro software. Intensity of markers on each numbered cells were plotted in the matched numbered plots and single-stained IHC image of CD68 and CD206 from the same section. Red circle: CD68+CD206++ TAMs, Green square: CD68+CD206+ TAMs. X axis: Distance in pixels, Y axis: Intensity of marker. Colors in the plots: CD206 (red), CD68 (green) and DAPI (blue). Scale bar: 100 μm. **g** Quantification of the maximum intensity of CD68 and CD206 on the macrophages (n=20 for each population) in Supplementary Fig 1f. Error bars represent mean ± SD, ***p<0.001. Mann–Whitney's U test. Each point represents one patient.

Supplementary Figure 2

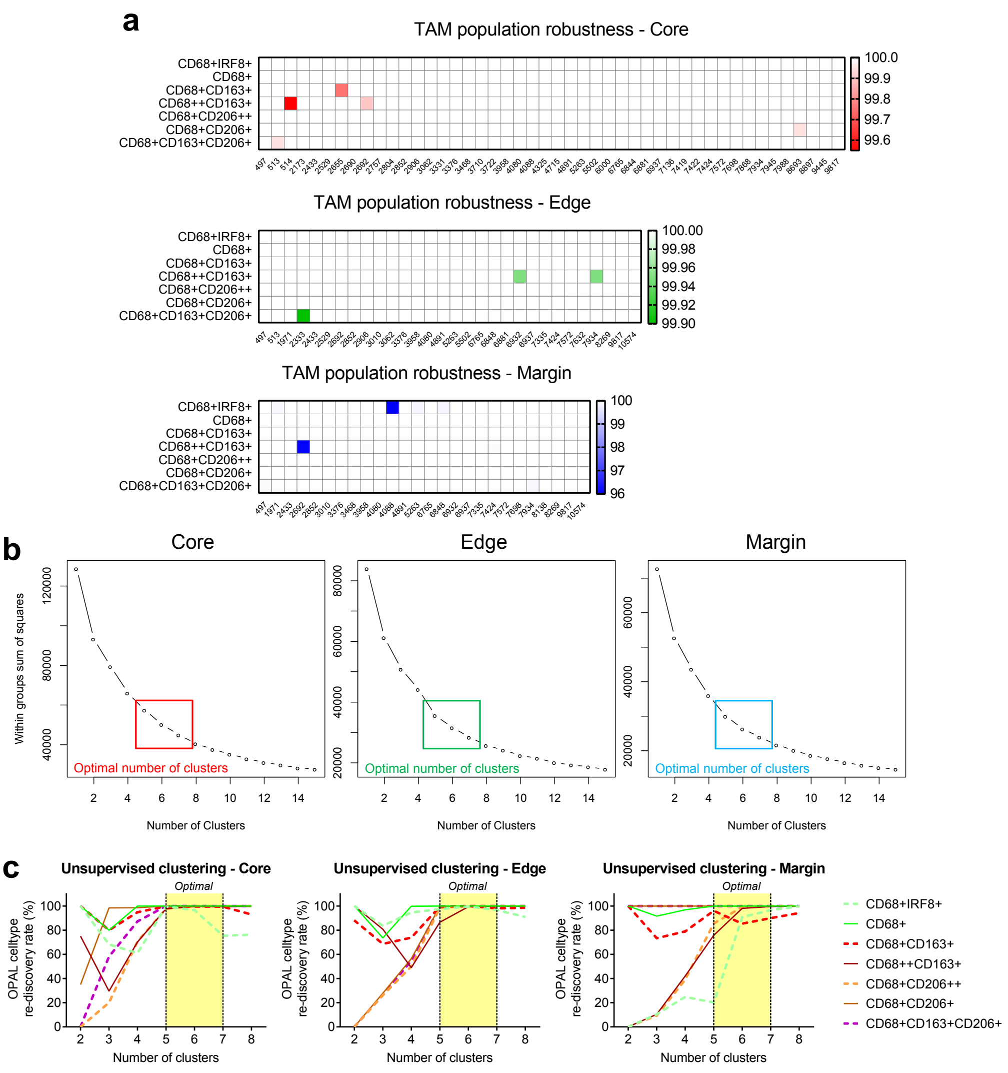

Supplementary Figure 2: Validation of TAM population subgrouping/clustering

a Heatmaps represent the accuracy of subsampling of TAMs from each of the TAM population per patient (bottom). Robustness of TAM subgrouping approach for each TAM population per patient in each ROI. 100 cells were randomly subsampled from each of the seven TAM populations per patient within each ROI (Core, Edge, and Margin). The subsampling was repeated for 2000 times. The median marker expression signatures of the subsample cohort (each TAM population in each patient per ROI was compared) to the signature of the overall cohort using the Pearson correlation ($p<0.05$ to test the statistical robustness of the subsampling. The accuracy of the subsampling of each TAM population was at least 96% accurate using the supervised inForm software. Scale: Accuracy (%) **b** Validation of TAM population clusters. The same subsampled cohort (n=500) were used to validate the number of populations using an unsupervised clustering method. The "within group sum of squares (wss)" for different ROIs using the K-means clustering method. Based on the "elbow method", the results suggest that for all ROIs, the optimal number of clusters was around 5 to 7 with our panel. **c** The median marker expression signatures from each clusters with the expression signature of the TAM populations to validate if the TAM populations can be rediscovered in the unsupervised clustering (Pearson, $p<0.01$). The results indicate that within the optimal clustering range (5-7), all TAM populations can be rediscovered, and were much better than other clustering numbers (2-4).

Supplementary Figure 3

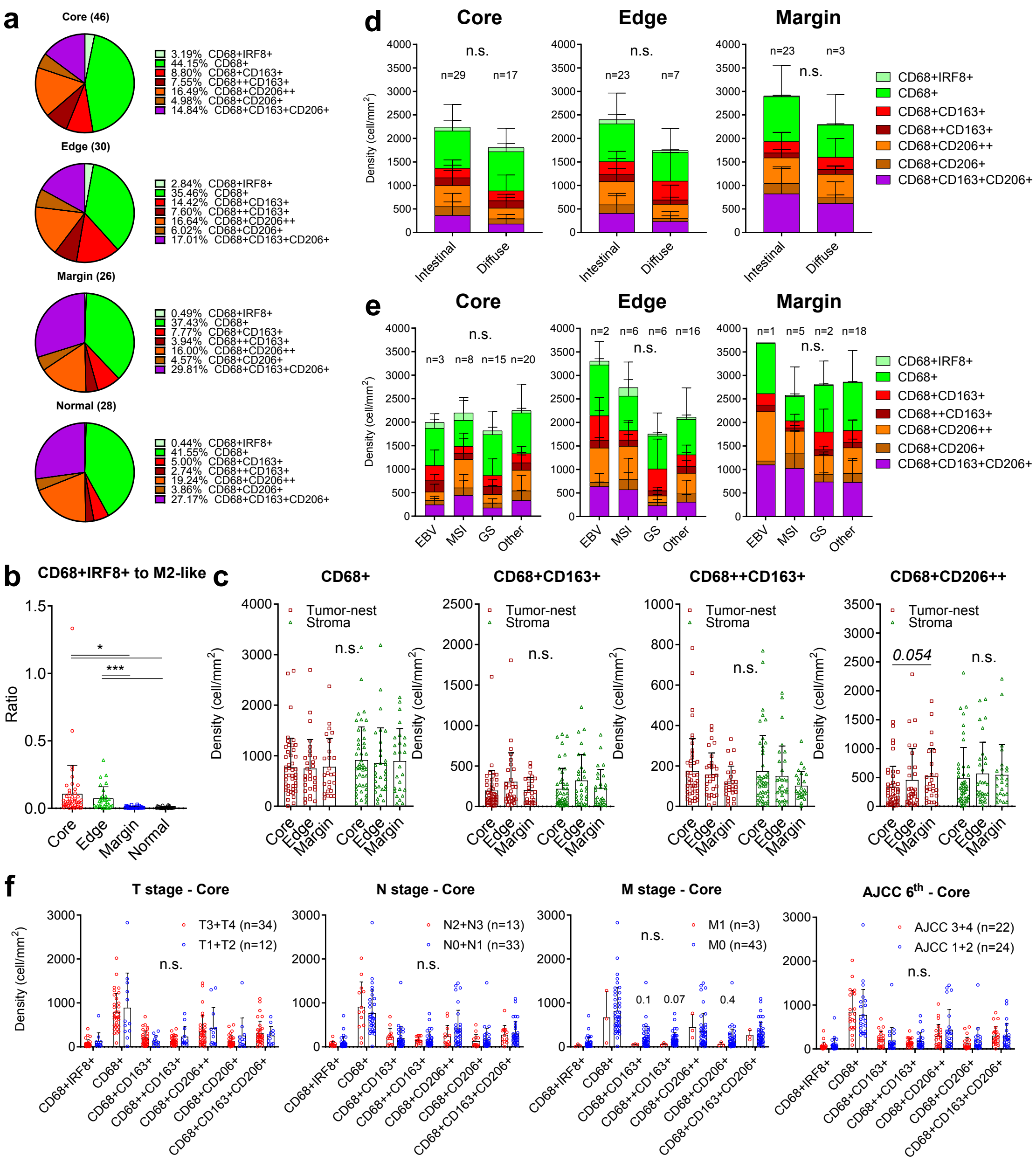

Supplementary Figure 3: Density of TAMs varies across regions of interest but not between different cancer subtypes
a TAM population percentage in each ROI. **b** CD68+IRF8+ to M2-like TAMs ratio among the ROIs. Error bars represent mean ± SD. Each dot represents one patient. *p<0.05, ***p<0.001, and not significant (n.s.). Mann–Whitney's U test. **c** Distribution of macrophage populations in the tumor-nest and stoma areas. Mean ± SD. Each dot represents onepatient. Not significant (n.s.). Mann–Whitney's U test. **d-f** Constitution of TAM populations in patient at different ROIs characterized with (**d**) Lauren histological subtype, (**e**) TCGA molecular subtype and (**f**) clinical staging. Error bars represent mean ± SD. Each dot represents one patient. Not significant (n.s.). Mann–Whitney's U test.

Supplementary Figure 4

a

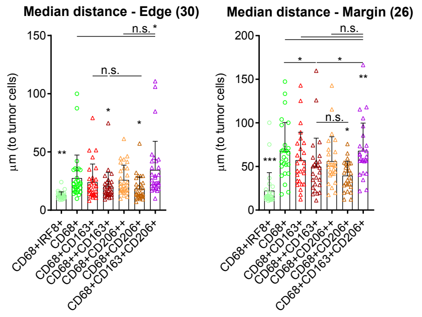

b

#	0-10 μm	Effective percentage (%) Median	Average	SD
1	CD68+IRF8+	26.75	31.23	12.00
2	CD68+	15.53	19.08	11.51
3	CD68+CD163+	23.97	25.23	15.15
4	CD68++CD163+	18.69	20.20	11.13
5	CD68+CD206++	15.89	19.84	13.10
6	CD68+CD206+	31.21	33.11	13.58
7	CD68+CD163+CD206+	12.48	13.86	10.20
	10-20 μm			
1	CD68+IRF8+	47.27	46.96	8.64
2	CD68+	29.90	31.16	7.55
3	CD68+CD163+	31.52	31.23	8.07
4	CD68++CD163+	40.09	39.28	8.43
5	CD68+CD206++	32.04	31.30	9.20
6	CD68+CD206+	32.47	32.63	5.00
7	CD68+CD163+CD206+	31.41	31.03	10.41

c

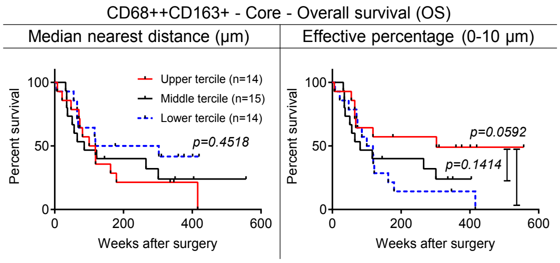

d

Overall survival (OS) - Effective density (0-10μm; cell/mm^2) - Core

CD68++CD163+ (RC) to tumor cells (NC)

Terciles	Cancer Subtypes: Intestinal	Diffuse +Mixed	EBV +MSI	GS +Other	TNM Stages: T4 +T3	T2 +T1	N3 +N2	N1 +N0	AJCC: 4 +3	2 +1	Median Density
Upper	10	4	6	8	8	6	3	11	5	9	54.30
Middle	10	5	2	13	11	4	5	10	8	7	22.71
Lower	7	7	1	13	12	2	4	10	8	6	7.15
Chi-square	0.4668		0.0451		0.2396		0.7731		0.4788		- -

e

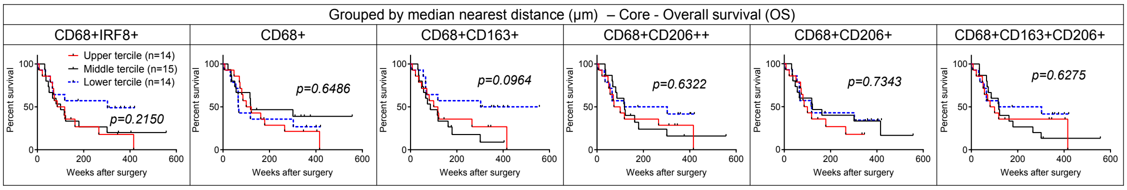

f

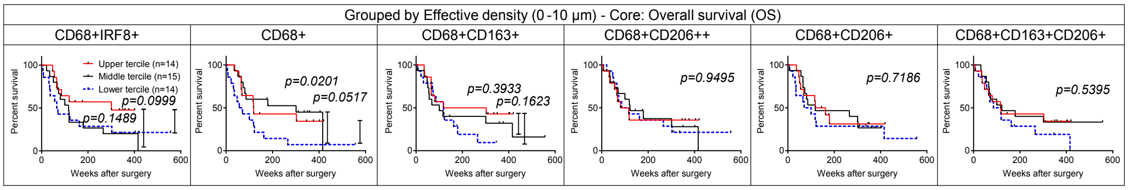

Supplementary Figure 4: TAM-composition change is associated with their proximity to tumor cells
a Median distance of TAM populations (RC) to tumor cell (NC) at the edge and margin. Error bars represent mean ± SD. Each dot represents one patient. *p<0.05, **p<0.01, ***p<0.001 and not significant (n.s.). Mann–Whitney's U test. Light green: CD68+IRF8+, Green: CD68+, Red: CD68+CD163+, Dark red: CD68++CD163+, Orange: CD68+CD206++, Brown: CD68+CD206+, Purple: CD68+CD163+CD206+. Circle: M1-like macrophages. Triangle: M2-like macrophages. **b** Effective percentage of TAM populations within 0-10 and 10-20 μm. **c** Patient OS classified with the median nearest distance and effective percentage (0-10 μm) of CD68++CD163+ TAMs (RC) to tumor cells (NC) in the core. Red: Upper tercile (density >2/3 of the patients in the study cohort), Black: Middle tercile (2/3 ≥ density > 1/3), Blue: Lower tercile (density ≤ 1/3). Log-rank (Mantel-Cox) test. **d** Univariate analyses of clinical parameters to CD68++CD163+ TAM classified OS. Chi-square analysis. **e-f** TAM classified patient OS using the (**e**) median nearest distance and (**f**) effective density (0-10 μm) of each TAM population (RC) to tumor cell (NC) in the core.

Supplementary Figure 5

a

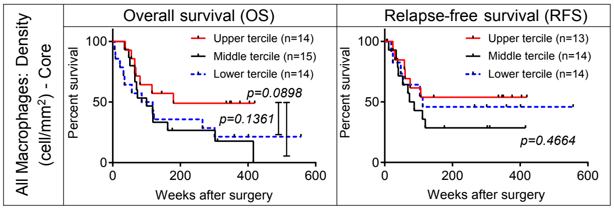

Median density (cell/mm²) - Core	All Macrophage		
	Terciles	OS	RFS
	Upper	2785.11	2895.27
	Medium	1718.16	1839.13
	Lower	1172.18	1172.18

b

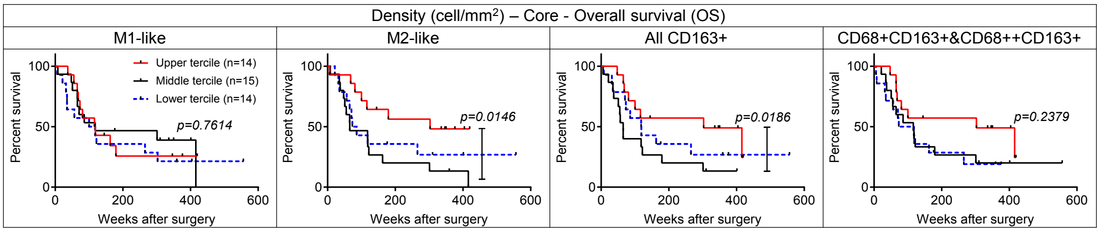

c

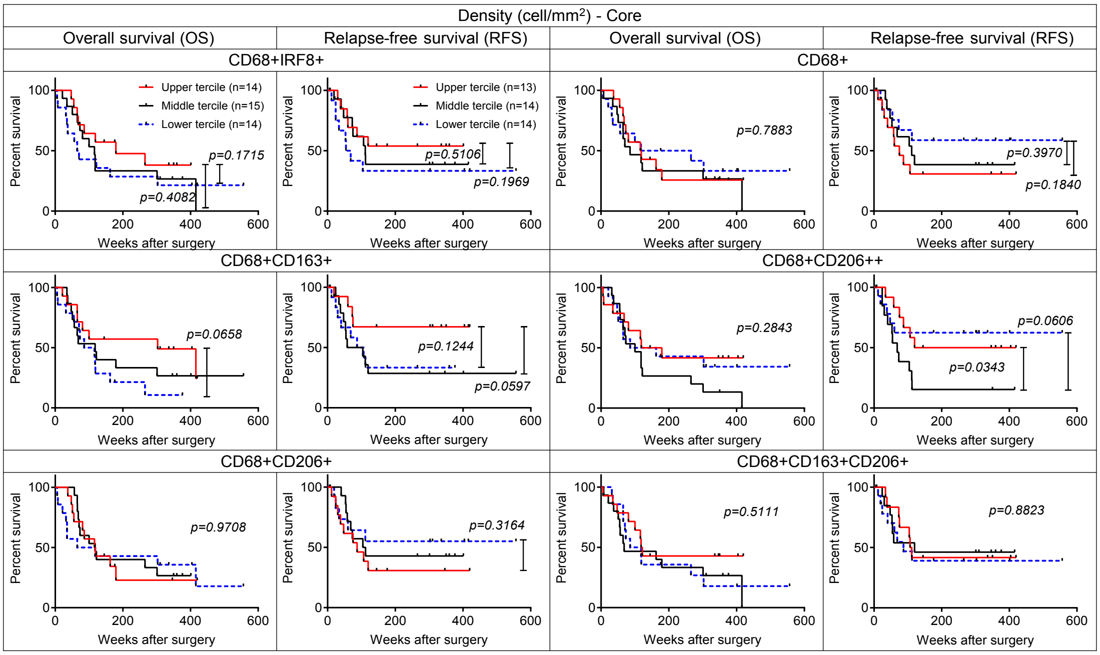

d

Density (cell/mm²) – Core		Cancer subtypes				TNM stages				AJCC stages		
		Overall Survival (OS)										
	Terciles	Intestinal	Diffuse +Mixed	EBV +MSI	GS +Others	T4+T3	T2+T1	N3+N2	N1+N0	4+3	2+1	Median density
CD68++ CD163+	Upper	9	5	4	10	10	4	4	10	6	8	254.14
	Medium	9	6	3	12	11	4	5	10	8	7	117.80
	Lower	9	5	2	12	10	4	3	11	7	7	60.76
	Chi-square	0.9623		0.6456		0.9912		0.7731		0.8482		- -
		Relapse-free survival (RFS)										
	Terciles	Intestinal	Diffuse+Mixed	EBV+MSI	GS+Others	T4+T3	T2+T1	N3+N2	N1+N0	4+3	2+1	Median density
	Upper	9	4	4	9	9	4	3	10	5	8	254.66
	Medium	8	6	3	11	10	4	6	8	8	6	116.42
	Lower	9	5	2	12	10	4	3	11	7	7	60.76
	Chi-square	0.9623		0.6456		0.9912		0.7731		0.8482		- -

e

	Coefficients	Standard Error	t Stat	P-value
Age	-0.1145	0.0487	-2.3488	0.0246
T.Stage	0.3435	0.1202	2.8582	0.0071
N.Stage	0.3603	0.1460	2.4682	0.0186
M.Stage	0.2669	0.2248	1.1874	0.2431
AJCC[6th]	-0.2682	0.1471	-1.8236	0.0768
TCGA	-0.5830	0.1275	-4.5720	0.0001
CD68++CD163+ density	-0.0921	0.0684	-1.3456	0.1871

Supplementary Figure 5: Different TAM groupings have minimal effects on patient survival
a-c Patient overall survival (OS) and relapse-free survival (RFS) classified using density of (**a**) all macrophages, (**b**) subgroupings of M1-like and M2-like TAMs and (**c**) the seven distinct TAM population identified in the study. Red: Upper tercile (density >2/3 of the patients in the study cohort), Black: Middle tercile (2/3 ≥ density > 1/3), Blue: Lower tercile (density ≤ 1/3). Log-rank (Mantel-Cox) test. **d** Univariate analysis of clinical parameters to CD68++CD163+ TAM density. Chi-square analysis. **e** Multivariate analysis of clinical parameters and CD68++CD163+ TAM density. Linear regression.

Supplementary Figure 6

a

b

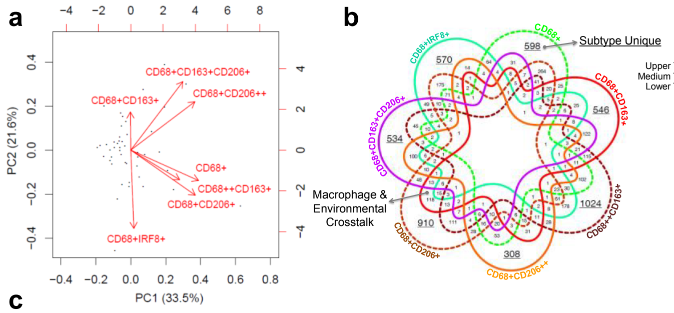

d

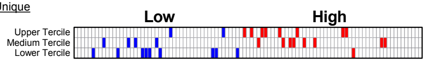

		MAUGIC Low	High	Accuracy	Overall
OPAL Terciles	Upper	2	8	80%	**75%**
	Medium (L-H)	4 (2-2)	8 (3-5)	60%	
	Lower	9	1	90%	

c

		Pathway name	FDR	Submitted entities found (*Italic*: gene names)												
Unique	POS	CD68+CD163+														
		Nonsense-mediated Decay (NMD) enhanced by the EJC	0.002	*NCBP2*	*RPL15*	*RPL19*	*RPL31*	*RPL34*	*RPL35A*	*RPL9*	*RPS18*	*RPS25*	*RPS27*	*RPS6*	*RPS9*	*UPF3A*
		SRP-dependent Cotranslational Targeting to Membrane	0.006	*RPL15*	*RPL19*	*RPL31*	*RPL34*	*RPL35A*	*RPL9*	*RPS18*	*RPS25*	*RPS27*	*RPS6*	*RPS9*	*SSR1*	
		Cap-dependent Translation Initiation / Completion	0.019	*RPL15*	*RPL19*	*RPL31*	*RPL34*	*RPL35A*	*RPL9*	*RPS18*	*RPS25*	*RPS27*	*RPS6*	*RPS9*		
		SHC-mediated Cascade:FGFR1	0.019	*FGF2*	*FGF9*	*FGFR1*	*SHC1*									
		Transcriptional Activation of p53 Response	0.046	*CDKN1A*												
		CD68++CD163+														
		Extracellular Matrix (ECM) Organization	0.000	*ADAMTS2*	*ADAMTS3*	*ASPN*	*BGN*	*CAPN3*	*CAPN6*	*COL14A1*	*COL1A2*	*COL3A1*	*COL4A1*	*COL5A2*	*COL6A3*	*COL8A1*
				CTSG	*CTSK*	*CTSL*	*CTSO*	*DCN*	*EFEMP1*	*FBLN1*	*FBLN2*	*FBLN5*	*FBN1*	*ITGB2*	*LAMA1*	*LAMA2*
				LAMA4	*LAMB1*	*LOX*	*LOXL3*	*LUM*	*MMP14*	*MMP9*	*NID2*	*PCOLCE*	*TGFB3*	*THBS1*	*TIMP1*	*VCAN*
				VWF												
		Interleukin-10 Signalling	0.026	*CCL4*	*CCL5*	*CCR1*	*CCR5*	*CD86*	*IL10RA*	*TIMP1*						
		MET Activated PTK2 Signaling	0.032	*COL1A2*	*COL3A1*	*COL5A2*	*LAMA1*	*LAMA2*	*LAMA4*	*LAMB1*						
		Nuclear Receptor Transcription Pathway	0.042	*ESR1*	*NR1H3*	*NR3C1*	*PPARD*									
		CD68+CD163+CD206+														
		HSF1-dependent Transactivation	0.021	*COL4A6*	*HSPA1L*	*HSPA2*										
		RUNX1 and FOXP3 Control the Development of Tregs	0.042	*CR1*	*IL2RA*											

		Pathway name	FDR	Submitted entities found (*Italic*: gene names)												
Crosstalk	POS	CD68+IRF8+														
		IGF2 mRNA Binding Proteins	0.021	*CD44*												
		CD68+														
		IGF2 mRNA Binding Proteins	0.001	*CD44*	*ZBP1*											
		CD68++CD163+														
		Signalling by Interleukins	0.018	*BCL2*	*CASP1*	*CSF3R*	*FLT3*	*FPR1*	*HGF*	*HMOX1*	*IL17RA*	*IL18BP*	*IL18RAP*	*IL1R1*	*IL23R*	*IL3RA*
				IL6ST	*ITGAX*	*JAK1*	*LCP1*	*NFKB1*	*RORA*	*S1PR1*	*SOCS2*	*STAT2*	*STAT5B*	*VIM*		
		IGF2 mRNA Binding Proteins	0.022	*CD44*												
		CD68+CD206++														
		IGF2 mRNA Binding Proteins	0.000	*CD44*												
		CD68+CD206+														
		Signalling by Interleukins	0.001	*CASP1*	*CSF3R*	*HMOX1*	*HNRNP*	*IGHG1*	*IL18BP*	*IL1R1*	*IL1R2*	*IL1RN*	*IL6R*	*IL6ST*	*IRAK2*	*ITGAX*
				JAK1	*LCP1*	*LYN*	*MAP2K7*	*MAPK8*	*PELI1*	*PSME4*	*PTPRZ1*	*RORA*	*S1PR1*	*SOCS2*	*STAT2*	*VRK3*
		Interleukin-6 signaling	0.009	*IL6R*	*IL6ST*	*JAK1*										
	NEG	CD68+CD163+														
		Polo-like kinase mediated events	0.000	*CCNB1*	*CCNB2*	*CENPF*	*LIN9*									
		Cell Cycle, Mitotic	0.001	*BIRC5*	*CCNB1*	*CCNB2*	*CDT1*	*CENPF*	*CENPN*	*CEP152*	*CEP70*	*CEP78*	*GINS2*	*GINS4*	*H2AFZ*	*HIST1H3A*
				HIST1H3I	*HIST1H4A*	*KIF2C*	*KMT5A*	*LIN9*	*NUP107*	*NUP155*	*NUP37*	*ORC4*	*SPDL1*	*TMPO*	*TOP2A*	
		Mitotic Prophase	0.018	*CCNB1*	*CCNB2*	*H2AFZ*	*KMT5A*	*NUP107*	*NUP155*	*NUP37*	*TMPO*	*HIST1H3A*	*HIST1H3I*	*HIST1H4A*		
		Signaling by FGFR2 IIIa TM	0.020	*FGFR2*												
		M Phase	0.045	*BIRC5*	*CCNB1*	*CCNB2*	*CENPF*	*CENPN*	*CEP152*	*CEP70*	*CEP78*	*H2AFZ*	*HIST1H3A*	*HIST1H3I*	*HIST1H4A*	*KIF2C*
				KMT5A	*NUP107*	*NUP155*	*NUP37*	*SPDL1*	*TMPO*							
		CD68++CD163+														
		TP53 Regulated DNA Repair	0.001	*CCNT1*	*CDK12*	*CHEK1*	*FANCD2*	*FANCI*	*GTF2H1*	*MDC1*	*POLR2L*					

e

f

g

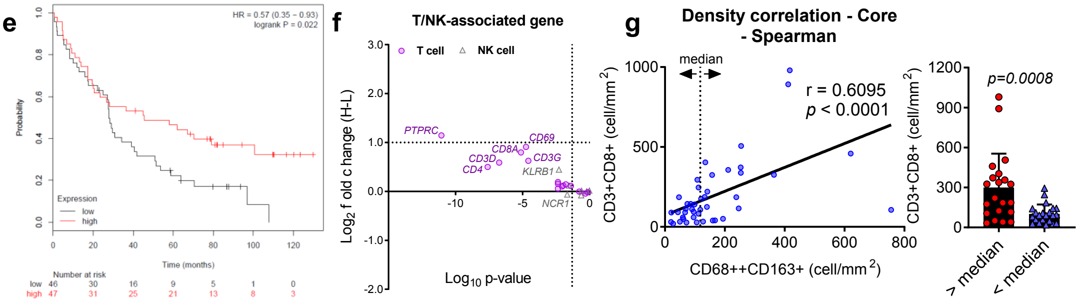

Supplementary Figure 6: Mixture of TAM populations within a same milieu

a Principle component analyses (PCA) of the density of TAM population in the core. **b** Venn diagram of the environmental gene signature for each TAM population, highlighting the population unique and shared crosstalk regions (number of genes).

c Pathways significantly associated with population unique and shared crosstalk genes of each TAM population. Italic: gene names. **d** Method of validation for the correlation between IHC and transcriptomic data. Patients who had both tumor core region for IHC and microarray data available were selected (n=34) for the generation of a CD68++CD163+ TAM associated signature (Fig. 4c). Patients in the MAUGIC cohort (n=99) were characterized into two signature-expressing groups. Patients (n=32 of the 34, the 2 removed from survival analysis were associated with surgical death) who were included in the overall survival analyses for the multiplex IHC study were selected and validated by comparing the relative groupings between the two methods. Patients grouped as the gene signature-high or low group as well as in the Upper or Lower tercile were categorized as accurate grouping, respectively. Patients in the Medium tercile were divided into two groups with the median density of the IHC staining cohort (n=34) and then tested for accuracy. Red/blue: number of patients grouped into the High/Low groups using the IHC derived cell density. **e** Patient OS of the MAUGIC cohort classified using the CD68++CD163+ TAM derived environmental signature. Log-rank (Mantel-Cox) test. **f** Differentially expressed T and NK cell associated genes between groups. **g** Correlation of CD68++CD163+ TAM and CD3+CD8+ T cell density in the Core and comparison between two density groups. Error bars represent mean ± SD. Each dot represents one patient. Mann–Whitney's U test.

Supplementary Figure 7

a

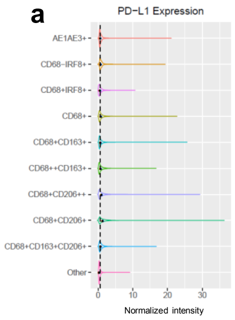

c

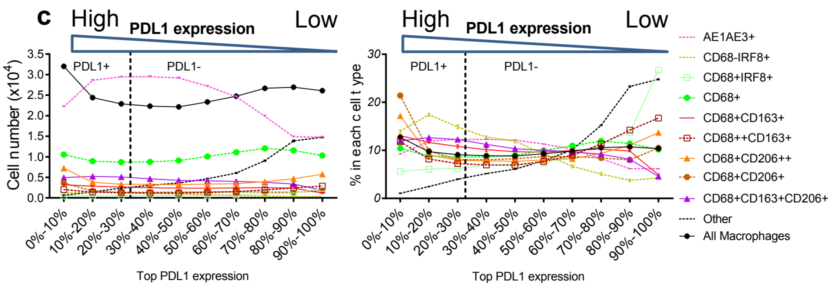

b

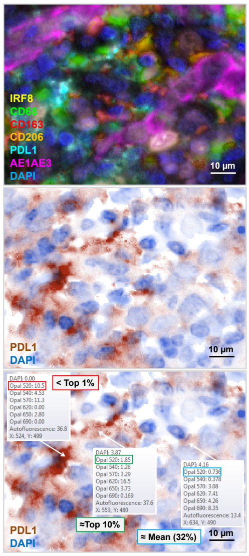

e

Median PDL1 Expression on TAMs		Cancer subtype				TNM stages				AJCC stages	
	Terciles	Intestinal	Diffuse+Mixed	EBV+MSI	GS+Others	T4+T3	T2+T1	N3+N2	N1+N0	4+3	2+1
CD68+ IRF8+	Upper	12	2	6	8	11	3	2	12	7	7
	Medium	7	8	1	14	9	6	5	10	6	9
	Lower	8	6	2	12	11	3	5	9	8	6
	Chi-square	0.0817		0.0432		0.4329		0.3801		0.6496	
CD68+	Terciles	Intestinal	Diffuse+Mixed	EBV+MSI	GS+Others	T4+T3	T2+T1	N3+N2	N1+N0	4+3	2+1
	Upper	12	2	6	8	10	4	4	10	7	7
	Medium	11	4	2	13	11	4	3	12	7	8
	Lower	4	10	1	13	10	4	5	9	7	7
	Chi-square	0.0043		0.0451		0.9912		0.6397		0.9785	
CD68+ CD163+	Terciles	Intestinal	Diffuse+Mixed	EBV+MSI	GS+Others	T4+T3	T2+T1	N3+N2	N1+N0	4+3	2+1
	Upper	12	2	6	8	10	4	3	11	6	8
	Medium	10	5	2	13	12	3	5	10	9	6
	Lower	5	9	1	13	9	5	4	10	6	8
	Chi-square	0.0219		0.0451		0.6397		0.7731		0.563	
CD68++ CD163+	Terciles	Intestinal	Diffuse+Mixed	EBV+MSI	GS+Others	T4+T3	T2+T1	N3+N2	N1+N0	4+3	2+1
	Upper	12	2	6	8	10	4	3	11	6	8
	Medium	10	5	2	13	12	3	5	10	10	5
	Lower	5	9	1	13	9	5	4	10	5	9
	Chi-square	0.0219		0.0451		0.6397		0.7731		0.2151	
CD68+ CD206++	Terciles	Intestinal	Diffuse+Mixed	EBV+MSI	GS+Others	T4+T3	T2+T1	N3+N2	N1+N0	4+3	2+1
	Upper	13	1	6	8	10	4	3	11	6	8
	Medium	9	6	2	13	11	4	6	9	10	5
	Lower	5	9	1	13	10	4	3	11	5	9
	Chi-square	0.0072		0.0451		0.9912		0.4329		0.2151	
CD68+ CD206+	Terciles	Intestinal	Diffuse+Mixed	EBV+MSI	GS+Others	T4+T3	T2+T1	N3+N2	N1+N0	4+3	2+1
	Upper	13	1	6	8	12	2	4	10	8	6
	Medium	9	6	2	13	10	5	3	12	8	7
	Lower	5	9	1	13	9	5	5	9	5	9
	Chi-square	0.0072		0.0451		0.3801		0.6397		0.4788	
CD68+ CD163+ CD206+	Terciles	Intestinal	Diffuse+Mixed	EBV+MSI	GS+Others	T4+T3	T2+T1	N3+N2	N1+N0	4+3	2+1
	Upper	12	2	6	8	11	3	3	11	7	7
	Medium	9	6	2	13	11	4	5	10	9	6
	Lower	6	8	1	13	9	5	4	10	5	9
	Chi-square	0.0614		0.0451		0.695		0.7731		0.423	

d

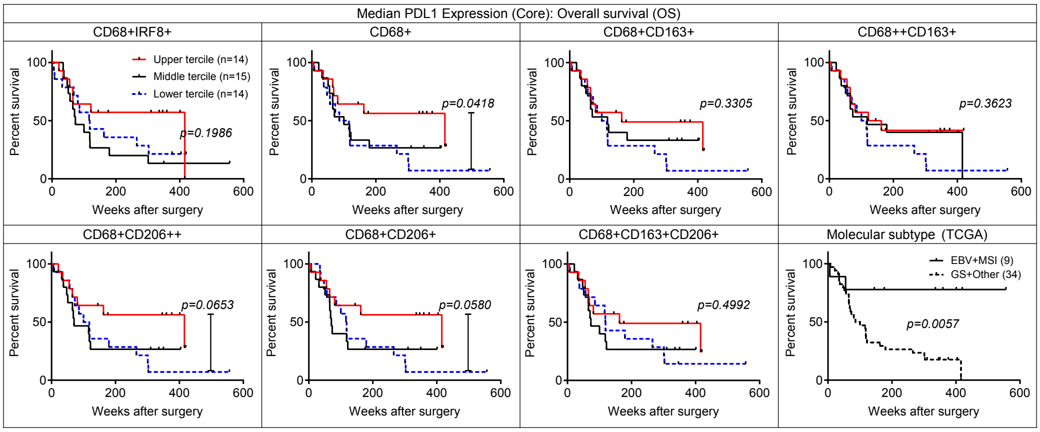

Supplementary Figure 7: PDL1 expression is both TAM and gastric cancer subtype associated
a Normalized PDL1 intensity on each cell type. Dashed line: mean intensity of all cell types, black dots: average expressions of the individual cell types. **b** Representative images of PDL1+ cells and intensity of its ranking. **c** Cell number and percentage of PDL1+ cells in each cell type per interval (from high [0%] to low [100%]). Dot and error bar show mean ± SD of five independent randomly selected sampling cohorts (n=5). Dashed line shows the mean PDL1 intensity of the cohort which was used to define the positivity of PDL1. Error bars represent mean ± SD of five independent randomly selected subsampling cohorts. **d** TAM classified patient OS using median PDL1 expression on each TAM population and the survival classified with cancer molecular (TCGA) subtype. Upper tercile (density >2/3 of the patients in the study cohort), Black: Middle tercile (2/3 ≥ density > 1/3), Blue: Lower tercile (density ≤ 1/3). Log-rank (Mantel-Cox) test. **e** Univariate analyses of clinical parameters of TAM population classified OS in Supplementary Fig. 7d. Chi-square analysis.

Supplementary Figure 8

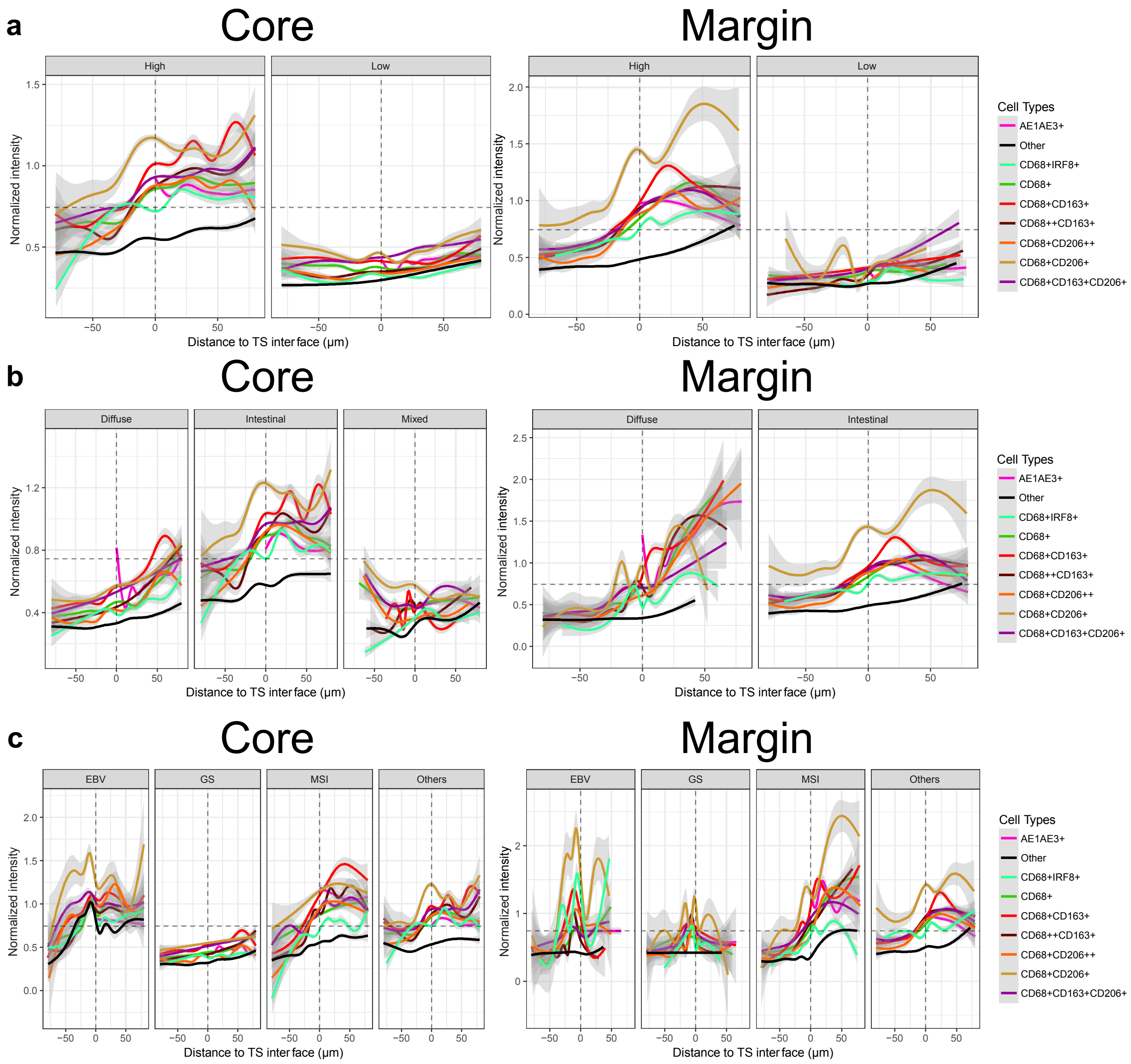

Supplementary Figure 8: High PDL1 TAMs are located in the tumor-nest areas and are gastric cancer subtype associated

a PDL1 expression on each cell population of patient defined as PDL1-high (H) and PDL1-low (L) in the core and at the margin. **b-c** PDL1 expression on each cell population of patient grouped by the cancer (**b**) Histological (Lauren) and (**c**) Molecular (TCGA) classification in the core and at the margin.
