# Supplementary material for: Macrophage spatial heterogeneity in gastric cancer defined by multiplex immunohistochemistry
Source: Nat Commun. 2019 Sep 2;10:3928. doi: 10.1038/s41467-019-11788-4 (PMC6718690; doi:10.1038/s41467-019-11788-4)
Supplement: Supplementary file 3 — Reporting Summary [file 41467_2019_11788_MOESM3_ESM.pdf]

nature research

Corresponding author(s): Alex Boussioutas

Last updated by author(s): Jul 16, 2019

Reporting Summary

Nature Research wishes to improve the reproducibility of the work that we publish. This form provides structure for consistency and transparency in reporting. For further information on Nature Research policies, see Authors & Referees and the Editorial Policy Checklist.

Statistics

For all statistical analyses, confirm that the following items are present in the figure legend, table legend, main text, or Methods section.

n/a	Confirmed	
☐	☒	The exact sample size (*n*) for each experimental group/condition, given as a discrete number and unit of measurement
☐	☒	A statement on whether measurements were taken from distinct samples or whether the same sample was measured repeatedly
☐	☒	The statistical test(s) used AND whether they are one- or two-sided<br>*Only common tests should be described solely by name; describe more complex techniques in the Methods section.*
☒	☐	A description of all covariates tested
☐	☒	A description of any assumptions or corrections, such as tests of normality and adjustment for multiple comparisons
☐	☒	A full description of the statistical parameters including central tendency (e.g. means) or other basic estimates (e.g. regression coefficient) AND variation (e.g. standard deviation) or associated estimates of uncertainty (e.g. confidence intervals)
☒	☐	For null hypothesis testing, the test statistic (e.g. *F*, *t*, *r*) with confidence intervals, effect sizes, degrees of freedom and *P* value noted<br>*Give P values as exact values whenever suitable.*
☒	☐	For Bayesian analysis, information on the choice of priors and Markov chain Monte Carlo settings
☒	☐	For hierarchical and complex designs, identification of the appropriate level for tests and full reporting of outcomes
☒	☐	Estimates of effect sizes (e.g. Cohen's *d*, Pearson's *r*), indicating how they were calculated

Our web collection on statistics for biologists contains articles on many of the points above.

Software and code

Policy information about availability of computer code

Data collection	Vectra microscope operation software, and Phenochart (version 1.0.8) software from PerkinElmer
Data analysis	R/R studio (version 3.3.1 for Windows). inForm (version 2.3.0) from PerkinElmer. ImagePro (Media Cybernetics). Distance analysis package (ISAT) used in this study is available online (Reference 37).

For manuscripts utilizing custom algorithms or software that are central to the research but not yet described in published literature, software must be made available to editors/reviewers. We strongly encourage code deposition in a community repository (e.g. GitHub). See the Nature Research guidelines for submitting code & software for further information.

Data

Policy information about availability of data

All manuscripts must include a data availability statement. This statement should provide the following information, where applicable:

- Accession codes, unique identifiers, or web links for publicly available datasets
- A list of figures that have associated raw data
- A description of any restrictions on data availability

Source data for figures [Figure 1a-h; Figure 2; Figure 3b-f; Figure 4a, c, e, f; Figure 5a, c-f, Figure 6] are provided with the paper. Microarray data is available online (GSE51105; https://www.ncbi.nlm.nih.gov/geo/query/acc.cgi?acc=GSE51105). Other data that support the findings of this study are available from the corresponding author [A.B.] upon reasonable request.

Field-specific reporting

Please select the one below that is the best fit for your research. If you are not sure, read the appropriate sections before making your selection.

☒ Life sciences ☐ Behavioural & social sciences ☐ Ecological, evolutionary & environmental sciences

For a reference copy of the document with all sections, see nature.com/documents/nr-reporting-summary-flat.pdf

Life sciences study design

All studies must disclose on these points even when the disclosure is negative.

Sample size

No sample size calculation was performed. The 56 samples used in the current study were selected which were representative of GC cancer subtypes, with detailed clinical information, and were multiplex IHC available (old and ethanol-fixed tissues were not ideal).

Data exclusions

Three patients with surgery-associated deaths (sepsis: 24days, pneumonia, cardiac arrest:13days, hyperkalaemia:65days) and two patients with no recurrence data documented were excluded for overall-survival and relapse-free survival analysis, respectively. The exclusion criteria were used to exclude surgical-related death as the study aim was to investigate the possible association of immune cell density to clinical outcome.

Replication

Subsampling of the TAMs from their own population were done to validate the robustness of the cell phenotyping by the inForm software. Clustering of TAM populations were validated using both supervised and unsupervised method (K-mean clustering). The robustness of the TAM subgrouping was above 96% across all tumor regions, repeated for 2000 times. See Supplementary Fig. 2.

Randomization

No randomization of patient was required in the study.

Blinding

The investigators were blinded while performing multiplex IHC, image acquisition and cell phenotyping analysis (raw-data collection). The investigators were not blinded afterwards in order to perform analyses on comparing different clinical parameters to determine their biological relevance.

Reporting for specific materials, systems and methods

We require information from authors about some types of materials, experimental systems and methods used in many studies. Here, indicate whether each material, system or method listed is relevant to your study. If you are not sure if a list item applies to your research, read the appropriate section before selecting a response.

Materials & experimental systems

n/a	Involved in the study	
☐	☒	Antibodies
☒	☐	Eukaryotic cell lines
☒	☐	Palaeontology
☒	☐	Animals and other organisms
☐	☒	Human research participants
☐	☒	Clinical data

Methods

n/a	Involved in the study	
☒	☐	ChIP-seq
☒	☐	Flow cytometry
☒	☐	MRI-based neuroimaging

Antibodies

Antibodies used

Multiplex IHC: IRF8 (Santa Cruz, E-9), CD68 (Leica Biosystems, 514H12), CD163 (Cell Marque, MRQ-26), CD206 (Abcam, Ab64693), PDL1 (Spring Bioscience, SP142) and multi-cytokeratin (Leica Biosystems, NCL-L-AE1/AE3). HRP-labelled anti-rabbit (PerkinElmer, NEF812001EA) and anti-mouse (PerkinElmer, NEF822001EA)
IHC: MLH1 (Leica, ES05), PMS2 (Ventana, EPR3947), MSH2 (Ventana, G219-1129) and MSH6 (BD Biosciences, 44)

Validation

Multiplex IHC: All antibodies were for human. IRF8 and CD206 can also be used for mouse. All primary antibodies were optimized using the positive control tissues as recommended by the manufacturers. IRF8 were optimized with healthy human tonsil tissues. CD68, CD163, CD206 were optimized with human liver tissue. PD-L1 was optimized with human placenta and the AE1AE3 was optimized with human skin tissue.
IHC: Antibodies are used routinely by the Pathology department at the Peter MacCallum Cancer Centre.

Human research participants

Policy information about studies involving human research participants

Population characteristics

See Supplementary Table 1

Recruitment	The patients were recruited for gastrectomy with curative intent. No biases associated with the result.
Ethics oversight	The Molecular Analysis of Upper Gastro-Intestinal Cancer (MAUGIC). All procedures were ethically approved by the Individual Review Boards (IRB) of the Peter MacCallum Cancer Centre and at each of the collection centers.

Note that full information on the approval of the study protocol must also be provided in the manuscript.

Clinical data

Policy information about clinical studies

All manuscripts should comply with the ICMJE guidelines for publication of clinical research and a completed CONSORT checklist must be included with all submissions.

Clinical trial registration	NA
Study protocol	NA
Data collection	NA
Outcomes	NA
